# The Relationship Between the Mediterranean Dietary Pattern and Exercise and Sport Performance—A Scoping Review

**DOI:** 10.3390/nu16244259

**Published:** 2024-12-10

**Authors:** Evangeline Mantzioris, Anthony Villani, Adrienne Forsyth

**Affiliations:** 1 Clinical & Health Sciences, Alliance for Research in Exercise, Nutrition and Activity (ARENA), University of South Australia, Adelaide, SA 5000, Australia; 2 School of Health, University of the Sunshine Coast, Sunshine Coast, QLD 4556, Australia; avillani@usc.edu.au; 3 School of Behavioural and Health Sciences, Australian Catholic University, Fitzroy, VIC 3065, Australia; adrienne.forsyth@acu.edu.au

**Keywords:** mediterranean diet, sports performance, athletes, anthropometrics, health

## Abstract

**Background/Objectives:** Athletes seek healthy diets for their health and performance. The Mediterranean Diet (MedDiet) has been widely studied for its health benefits. We conducted a scoping review of the scientific literature for studies reporting on the relationship between MedDiet adherence and performance and health outcomes in athletes. **Methods**: Five bibliographic databases were searched from inception to December 2023. We included studies with any competing, recreational, professional, elite, or occupational athlete (of any age) who played a physical sport as part of a team or as an individual, in which the MedDiet was used as an intervention, or adherence was measured. A total of 7993 unique records were identified, screened, and assessed for eligibility. **Results**: A total of 32 studies were included: 23 observational studies, 6 randomised controlled trials, and 3 quasi-experimental studies. Almost all studies (n = 31) were conducted in Mediterranean countries. Anthropometrics (n = 19) and body composition (n = 12) were the two most measured outcomes. There was a wide range of athlete ages and calibres and great variability in the outcomes reported; however, there were limited reports for each outcome. Only one RCT reported on specific sport-related performance outcomes. Three RCTs provided individualised dietary recommendations for macronutrients based on sports nutrition guidelines, but only two of them assessed macronutrient intake. **Conclusions**: This is the first scoping review of MedDiet adherence on outcomes related to sport. We found a limited evidence base across different sports and athletes, with few studies conducted outside of Mediterranean countries. Importantly, more intervention studies are needed to provide high-level causal evidence on the impact of the MedDiet pattern on performance and health outcomes in athletes.

## 1. Introduction

Athletes engage in a range of environmental and behavioural strategies to enhance performance [1,2,3]. Strategic, periodised, and individually tailored sports nutrition interventions have been shown to influence athletic performance [4]. In ideal situations, athletes work collaboratively with sports dietitians to develop and continuously improve their personal nutrition plan to achieve their sports performance goals while also considering their personal circumstances, preferences, and health [5]. However, even in elite sports settings, the opportunity for such personalised support may be limited [6]. Increasingly, athletes are using online sources of nutrition information and may lack the knowledge and skills to be able to identify and understand credible sources of nutrition information [7]. Athletes of all calibres typically have poor sports nutrition knowledge, and education interventions have shown minimal impact on nutrition knowledge and dietary intake [8,9,10]. Therefore, it may be helpful for athletes to have access to simple nutrition messages to inform a baseline diet that supports both health and athletic performance.

Researchers are encouraged to look to dietary patterns, combinations of food and drinks, and the frequencies at which they are habitually consumed before selecting individual foods and nutrients to study; however, sports nutrition guidelines have been built upon nutrient recommendations with more recent calls for a food-first approach [11]. It may therefore be prudent to consider taking a further step out to explore relevant and researched dietary patterns when considering broadly applicable dietary recommendations for athletes. The Mediterranean Diet (MedDiet) is one of the most well-known and widely studied dietary patterns worldwide [12,13,14]. While variations exist across the Mediterranean region, the research literature typically reflects the diet consumed in Greece and southern Italy before the mid-1960s [15]. In the research literature, the MedDiet is often described as a plant-based dietary pattern, consistent with a high intake of vegetables, fruits, nuts, legumes, and unprocessed cereals and the daily use of extra-virgin olive oil (EVOO) incorporated into all meals; a moderate consumption of fish, shellfish, fermented dairy products (cheese and yoghurt), and wine (typically during meals); and a low consumption of meat and meat products, processed cereals, sweets, vegetable oils, and butter [15,16]. A large and consistent body of evidence supports the MedDiet as being protective against chronic and inflammatory conditions [6,7,8,9,10,11,12,13,14,15,16,17,18], although the MedDiet’s application outside of the Mediterranean region, and to those with diverse backgrounds within the region, requires consideration [14,17,18]. The underlying mechanisms of protection are not completely understood, but one of the more compelling explanations relates to the antioxidant properties of the MedDiet [19,20]. Further, given that the MedDiet is predominately plant-based, the nutrient profile of the diet is such that it is naturally low in saturated fat and rich in several functional components, including vitamins and minerals, carotenoids, unsaturated fatty acids, and phenolic compounds, depicted by antioxidant and anti-inflammatory properties [21].

Recently, there has been increasing attention on the use of the MedDiet in sport [22,23,24,25,26,27,28,29]. Griffiths and colleagues have described the potential mechanisms through which the MedDiet may enhance sports performance in detail in their narrative review [29]. The potential pathways postulated to be involved in supporting exercise and sports performance have been reviewed extensively. Briefly, they include reductions in oxidative stress, anti-inflammatory effects, improving cognitive and vascular functioning during sports and exercise performance, and illness and injury prevention [29]. This has been attributed to the increased levels of polyphenols, nitrates, and omega-3 fatty acids that are in the predominately plant-based MedDiet which have been shown to improve exercise performance [30].

The impact of the MedDiet on sport and exercise performance has not been systematically evaluated. Thus, the aim of this scoping review was to examine the breadth and depth of the current literature on the MedDiet and outcomes related to sport and exercise performance in order to 1: synthesise and ascertain the scope of the current evidence; 2: identify gaps in the current literature to direct future research; and 3: provide recommendations to inform future research and practice.

## 2. Materials and Methods

The protocol for this scoping review was prospectively designed and registered with Open Science Framework (https://doi.org/10.17605/OSF.IO/U7WMP, accessed on 6 August 2021) with the results reported according to the Preferred Reporting Items for Systematic Reviews—Extension for Scoping Reviews (PRISMA-ScR) checklist [31].

### 2.1. Literature Search Strategy

An initial search was run on 6 August 2021 using PubMed, OvidEmcare, Scopus, Web of Science, and SportDiscus to search for articles from inception to the present date with the search terms “Mediterranean diet” AND (sport* OR athlet*) applied across all databases. Searches were limited to English-language articles in all databases and to human studies in PubMed, OvidEmcare, and Scopus (see Supplementary Table S1). The search was re-run and updated on 7 December 2023 using the same databases, search terms, and limits. The reference lists of the included articles were handsearched.

### 2.2. Eligibility Criteria

Included studies were full-text articles, published in peer-reviewed journals, and written in English. Both intervention (randomised controlled trials (RCTs), quasi-experimental studies) and observational (cohort, case–control, cross-sectional) studies were included. The population, intervention/exposure, comparison, outcomes, and study type (PICOS) framework used to develop the inclusion criteria, as outlined in Table 1. Reviews and studies published as letters to editors, editorials, conference abstracts, or posters were excluded.

### 2.3. Study Selection, Data Management, and Extraction

The articles retrieved from the databases were deduplicated in EndNote X9 (Clarivate, Philadelphia, PA, USA) then imported into Covidence (Veritas Health Innovation, Melbourne, Australia) for a two-stage screening process. First, two reviewers (two of EM, AV, BS, AF) independently screened titles and abstracts, and then in the second stage, full-text articles were assessed for eligibility. Discrepancies were resolved through discussion and consensus between EM and AV or by a third reviewer (AF), if required. Reviewers discussed the inclusion and exclusion criteria and jointly screened/assessed a sample of articles prior to each stage to ensure a common understanding.

A data extraction spreadsheet was jointly prepared by all authors. Data were extracted using Microsoft Excel (Microsoft Corporation, Washington, DC, USA) by two independent reviewers (EM, AV, AF, or BS). The data extracted were subsequently discussed among the authors to reach consensus. The data extracted included study design, subject characteristics (including athlete type, age, anthropometrics), setting, study aims, intervention characteristics, tools used to assess adherence to the MedDiet, and any outcomes related to sporting/exercise performance.

### 2.4. Analysis and Reporting

Due to the heterogeneous nature of the included studies and the exploratory nature of the study aim, a meta-analysis was not appropriate, and the results were narratively synthesised. The results presented include study characteristics and outcomes related to sports and exercise performance. Given the potential for the MedDiet to influence health, other clinical outcomes were also reported.

### 2.5. Quality Assessment

All studies were evaluated for quality using the Joanna Briggs Institute quality assessment tools for RCTs, cohort studies, and cross-sectional studies by two reviewers (two of EM, AV, AF, BS) [32]. Any conflicts were resolved through mutual discussion or by a third reviewer not involved in the initial screening, if required. As the quality of the reported MedDiet intervention adopted in RCTs posed a potential source of bias in this scoping review, the quality of the reported MedDiet interventions was assessed by two reviewers (EM and AV), using an independent assessment tool previously developed for use in systematic reviews [33], which contains 19 unique elements describing the traditional MedDiet pattern and cuisine. For each element, studies were assessed by the following three factors: prescribing the element, specifying the quantity, and meeting the minimum criterion for the MedDiet pattern. The assessment tool also considered the overall design and management of the dietary intervention and composition of the diets [33].

### 2.6. Equity, Diversity, and Inclusion Statement

Our research team included diversity across genders and cultural backgrounds and with representation from researchers at different career stages. In our review, we included all manuscripts from all types of athletes. In our synthesis of data, we identified and discussed gaps in research, including research on para and masters athletes.

## 3. Results

**Figure 1:**
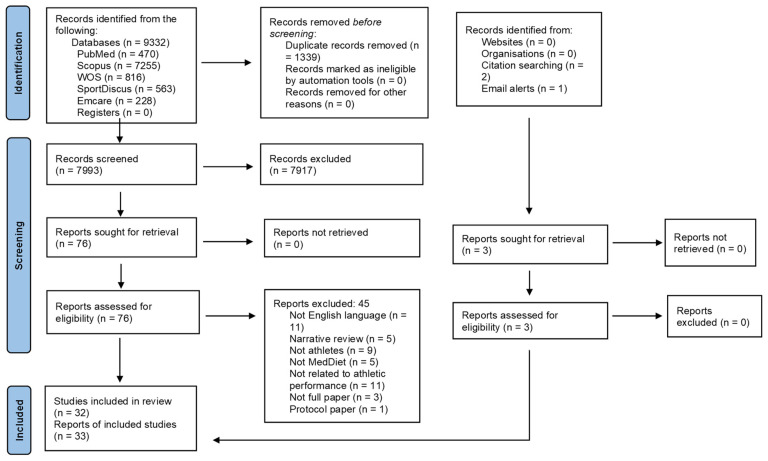
PRISMA flow diagram of the literature search process indicating the number of papers identified, screened, and included in the review.

The searches yielded 9332 citations; after the removal of 1339 duplicates, a total of 7993 articles were screened based on their title and abstract. Seventy-six articles were eligible for full-text assessment. Two were added from handsearching, and one was added from database email alerts. A total of 32 studies from 33 papers were deemed eligible for inclusion. Reasons for exclusion are shown in Figure 1.

Twenty-three observational studies (all cross-sectional) [34,35,36,37,38,39,40,41,42,43,44,45,46,47,48,49,50,51,52,53,54,55,56,57], six randomised controlled trials (RCTs) [58,59,60,61,62,63], and three quasi-experimental studies [64,65,66] (Table 2) were published between 2008 and 2023. All participants were healthy athletes with no reported injuries or illnesses and with a BMI between 18.5 and 25 kg/m^2^.

All observational studies (n = 23) were conducted with populations in the Mediterranean region, with seventeen in Spain [34,36,37,38,39,40,42,43,45,46,47,48,49,50,53,54,55], two in Greece [44,51], two in Italy [35,56], and one each in Portugal [52] and Croatia [57]. Most (n = 11) of these were conducted with younger athletes (e.g., children or adolescents) [34,36,37,38,40,45,46,47,51,52,54]. Only six observational studies were conducted with adults [35,43,53,56,57,67], and seven studies included both children and adults [39,42,44,48,49,50,55]. Half of the observational studies included both males and females [34,35,36,38,40,42,43,45,50,54,57], whereas four studies included females only [39,46,49,51], and a further eight included males only [37,44,47,48,52,53,55,56].

Five of the six RCTs were conducted in Italy or Spain [59,60,61,62,63], with one study conducted in the USA [58]. All six RCTs were conducted with adults, two studies included males and females [58,62], three included males only [59,60,61], and one study included females only [63].

The three quasi-experimental studies were also conducted in the Mediterranean region, including Cyprus [64], Italy [66], and Algeria [65]. Two of these studies included male and female adolescents [64,65]; the other included adult males [66].

The studies included athletes participating in a variety of sports including sailing [40,41], soccer [37,47,52], swimming [34,64], ultramarathon running [35], gymnastics [46,49,51], beach handball [39,42,48,55,63], cycling [43,44], tennis [45], kickboxing [61], volleyball [60,66], cross-fit [62], canoe polo [50], rugby [53], basketball [54], and wheelchair basketball [56]. Five studies categorised athletes based solely on their performance level and did not specify sport type [36,38,58,59,65]. Athletes of different calibres were equally represented in the included studies; thirteen studies were conducted on amateur athletes [23,44,45,46,49,51,58,59,60,61,62,64,65] and three on school/college athletes [36,37,38]. Studies involving higher-performance athletes described these athletes as elite-level athletes in five studies [39,40,47,48,50] and professional athletes in one study [57], and three studies described the athletes as nationally competitive [34,35,42,66].

A variety of tools and a priori scoring indices were used to assess adherence to the MedDiet. The KIDMED adherence tool was used in 16 of the observational studies [34,36,37,38,39,42,45,46,47,48,49,51,52,53,54,55] (Table 2). The Mediterranean Diet Adherence Screener (MEDAS) was used in three studies [40,43,50]. The Mediterranean Diet Scale (MDS) [35,57] and the Mediterranean diet serving score (MDSS) [44,56] were used each in two studies. Most of the observational studies reported moderate adherence to the MedDiet regardless of which adherence screeners they employed. Leao, 2023, reported high adherence to the MedDiet in soccer players from Portugal [52] and low adherence in canoe athletes in Spain [50]. 

Across the intervention studies, a combination of weighed food records [58,60,63] and food frequency questionnaires [59,60] were used to assess compliance with the MedDiet. The KidMed tool [64,65] and MEDAS [66] were used to assess adherence to the MedDiet, and three studies demonstrated increases in MedDiet adherence [58,64,65]. Analyses of the individual components of the MedDiet were conducted with adolescent swimmers from Greece who reported significant increases in the intake of EVOO and a decrease in the consumption of sweets and candy [64].

Gender differences were assessed in three studies; one reported higher adherence in female athletes compared to males [35]. Two observational studies [39,42] reported conflicting results between age and MedDiet adherence in beach handball players.

Three intervention studies [63,64,66] provided specific sports nutrition recommendations for energy or macronutrient (carbohydrate and/or protein) intakes for daily fuelling and for training and performance in agreement with either the American College of Sports Medicine (ACSM), International Society for Sports Nutrition, the International Olympic Committee (IOC) guidelines for athletes, or the recommendations for adolescents from Sports Dietitian Australia [4,68,69,70,71]. Two studies based fuelling-related dietary advice on the participants’ previous dietary intakes [58,61], and another recommended a ‘typical athlete meal’ according to the calculated energy needs of the athletes [65]. One study provided energy recommendations (based on the Harris–Benedict equation and an activity factor multiplier of 2.0) and protein recommendations (calculated according to the number of training sessions) [62]. The remaining two did not provide sports-specific nutrient recommendations [59,60].

**Table 2:** Characteristics of included studies exploring MedDiet adherence on any health- or sports performance-related outcomes.

Observational Studies					
Author Country of Study	Participants		MedDiet Measure and Adherence Level (Mean ± SD or %)	Outcomes	JBI Score for Quality of Cross-Sectional Study
Altavilla et al., 2021 [34] Spain	Semi-professional adolescent swimmers with minimum 3 years of experience and 5–6 swimming sessions (90–120 min, covering minimum 3000 m)/week m—n = 34, age 14.5 ± 1.3 years wt 56.5 ± 9.8 kg, BMI 20.06 ± 2.5 kg/m^2^ f—n = 40), age 13.6 ± 1.2 years wt 50.2 ± 8.1 kg, BMI 20 ± 2.8 kg/m^2^		KidMed m—8.09 ± 1.5 f—7.23 ± 2.2 (medium adherence)	BMI, Waist–Hip Ratio	6/8
Citarella et al., 2021 [35] Italy	Elite runners from Italian national ultramarathon team n = 10, age 41.1 ± 7.59 years, BMI 21.66 ± 1.11 kg/m^2^ m—n = 6, age 40.80 ± 5.4; BMI 21.79 ± 1.06 kg/m^2^ f—n = 4, age 39.5 ± 10.47, BMI 21.45 ± 1.31 kg/m^2^		MDAS Total 46.97 ± 11.96 m—39.94 ± 6.33 f—57.50 ± 10.78	Weight, Body composition, training volume (km per week), record in the 100 km competition	4/8
Kontele et al., 2021 [51] Greece	Adolescent female athletes who participated in all disciplines of gymnastics in Greece n = 269 btw age 11 and 18 years, mean age 13.89 ± 1.75 years, BMI 19.0 ± 2.45 kg/m^2^		KidMed 34.9% high adherence 56.1% moderate adherence 8.9% low adherence	Weight, BMI	6/8
Leao et al., 2023 [52] Portugal	Male soccer players Three training sessions/week of 90 min duration, and one competitive match/week n = 132, age 7–15 years		KidMed ave 8.36 ± 1.92 68.2% high, 31.1% moderate, 0.8% low adherence	BMI	7/8
Lopez-Jimenez et al., 2023 [53] Spain	Elite male rugby union players n = 35, age 24.8 ± 0.8 years, wt 99.2 ± 2.72 kg		KidMed Moderate adherence	Body weight, BMI	8/8
Manzano-Carrasco et al., 2020 [36] Spain	Athletes enrolled in different municipal school sports n = 1198; m—n = 875; and f—n = 323 age 6–17 years		KidMed m—35.3% high, 57.6% moderate, 7.1% low adherence f—33.1% high, 59.8% moderate, 7.1% low adherence	Body composition, 20 m shuttle run test, countermovement jump, forced vital capacity, forced expiratory volume, peak expiratory flow, mean forced expiratory flow	6/8
Manzano-Carrasco et al., 2020 [37] Spain	Adolescent male football players, enrolled in 3 municipal football sport schools, trained 2 d/w for at least 2 h n = 194, aged 8–16 years; average 12 ± 2 years		KidMed 7.14 ± 1.95	20 m shuttle run test, handgrip strength test	6/8
Manzano-Carrasco et. al., 2020 [38] Spain	Athletes who practice any sport modality at least 2 days a week for 1 h n = 1676, age 6–17 years; ave 11.11 ± 2.62 years wt 44.48 ± 15.25 kg, 56.6% of sample (n = 947) were classified as normoweight, 26.6% (n = 445) as overweight, and 16.9% (n = 284) as obese		KidMed 35.7% high, 57.6% medium, 6.7% low adherence	Weight, BMI, Body composition, 20mSRT, Abalakov jump, handgrip strength	6/8
Marques-Sule et al., 2022 [50] Spain	Professional canoe polo athletes n = 43 (m—36, f—5), n = 23 under 21 years, n = 20 over 21 years, mean age—21.54 ± 6.03 years BMI 23.27 ± 2.61 kg/m^2^		MEDAS-14, Healthy-Eating Index-Spanish 0% high, 30.2% moderate, 69.8% low adherence	Body fat; BMI; shoulder rotation range of motion; handgrip strength; push strength—overall; push strength—rowing; countermovement jump; push-up test; sit-to-stand test; motivation to exercise (Exercise Motivation index)	6/8
Martinez-Rodriguez et al., 2018 [40] & Martinez-Rodriguez et al., 2017 [41] Spain	Young elite sailors n = 75, m—n = 50, f—n = 25, age 15.7 ± 1.8 years		MEDAS n = 47 low adherence (<6 pts), n = 28 high adherence (>6 pts)	Sex, BMI, competition success, anxiety	8/8
Martinez-Rodriguez et al., 2021 [39] Spain	Female beach handball players (n = 33) on Spanish national team n = 18 juniors (Age 16.7 ± 0.50 years) N = 15 seniors (Age 24.8 ± 4.71 years) BMI (juniors): 22.5 ± 2.28 kg/m^2^ BMI (seniors): 22.8 ± 2.75 kg/m^2^		KidMed Junior—7.33 ± 1.61 (moderate adherence) Senior—6.27 ± 2.05 (moderate adherence)	Weight, BMI, handgrip test, Abalakov, yo-yo test, Bone Quality (Broadband ultrasound attenuation, sound of speed, and stiffness)	5/8
Martinez-Rodriguez et al., 2021 [42] Spain	Spanish national beach handball players, n = 59 Female n = 21, Junior: 16.1 ± 1.46 years; 14–17 years, wt—56.3 ± 8.7 kg, Senior: 23.2 ± 2.9 years; 18–28 years, wt—63.7 ± 8.9 kg Male n = 38, Junior: 17.0 ± 0.1 years; 15–18 years, wt—70.1 ± 11.2 kg, Senior: 25.5 ± 4.7 years; 18–35 years, wt—81.3 ± 7.6 kg		KidMed f—76% moderate, 9.52% low adherence m—66% moderate, 20% low adherence	BMI, Body composition, height, weight, handgrip strength, countermovement jump	6/8
Martinez-Rodriguez et al., 2022 [48] Spain	Professional male beach handball players n = 36, age 16–35 years, mean 20.9 ± 5.55 years Weight range 63.5–115 kg, mean 84.1 ± 14.0 kg BMI range 19.8–30.4 kg/m^2^, mean 24.6 ± 2.73 kg/m^2^		KidMed 6.78 ± 2.38 Junior—6.83 ± 2.26 Senior—6.72 ± 2.56	Body composition, weight, height, BMI, countermovement jump, Abalakov, handgrip strength, yo-yo test, VO_2_ max	6/8
Martinovic et al., 2022 [57] Croatia	Professional athletes n = 150, m—n= 58, BMI 24.8 ± 3.3 kg/m^2^, age 24.5 ± 4.0 year Recreational athletes n = 150—m 52 BMI 23.8 ± 3.0 kg/m^2^, age 24.0 ± 5.5 years		MDSS Professional athletes—24% high adherence Recreational athletes—14% high adherence	MET score, orthorexia nervosa (ORTO-15)	8/8
Mayolas-Pi et al., 2017 [43] Spain	Cyclists n = 859, m—751, and f—108, and inactive subjects n = 718, m = 307, and f—411		MEDAS Overall adherence not reported	Risk of exercise addiction	5/8
Morales-Suarez-Varela et al., 2023 [54] Spain	Basketball players, age 8–15 years n = 183, m—n = 107, f—n = 76		KidMed Mean scores between 8.71 ± 1.98 and 9.07 ± 1.48 across different groups	Athlete burnout questionnaire ABQ	6/8
Papdopoulou et al., 2017 [44] Greece	Male cycling athletes, age 15–50 years, with 6 years of sequential training and competed on Greek national team, n = 50, age 32 ± 20 years, BMI 23.65 ± 2.74 kg/m^2^		MedDiet Score n = 1 high, n = 43 moderate, n-6 low adherence	Physical performance	7/8
Pelaez-Barrios et al., 2022 [49] Spain	Acrobatic gymnasts N = 151 (N = 81 gymnasts and N = 70 non-gymnasts), age 10–19 years (mean 13.85 ± 2.45 years)		KidMed Gymnasts—70.4% high, 26.2% moderate, 2.5% low adherence Non-gymnasts—31.4% high, 62.9% moderate, 5.7% low adherence	Body shape questionnaire, height, weight, waist circumference, BMI, % body fat	6/8
Peraita-Costa et al., 2020 [45] Spain	Tennis players—children and adolescents, training minimum 2 h/day n = 94, age 8–15 years, n = 56 boys, and n = 38 girls		KidMed 78.7% moderate, 21.3% high adherence	Burnout syndrome	6/8
Romero-Garcia et al., 2022 [55] Spain	Male handball players from 12 to 28 years old n = 136, infant n = 35, 13.41 ± 0.4 year; cadets n = 46 14.83 ± 0.64 years and juniors n = 26 17.2 ± 0.55 years		KidMed Total—42.1% high, 47.4% moderate, 10.5% low adherence	Weight, height, Body composition, BMI, T-Half test, yo-yo test, VO_2_ max, squat jump, countermovement jump, overhead medicine ball throw test, 30 m sprint	6/8
Santana et al., 2019 [46] Spain	Female rhythmic gymnastics from 6 to 17 years old n = 221 (56.11% < 13 years)		KidMed 41.63% high, 52.94% medium, 5.43% low adherence	Waist circumference, BMI, height, weight	6/8
Santos-Sanchez et al., 2021 [72] Spain	71 U12 male soccer players Age 8–12 years (grouped as 8–10 and 10–12)		KidMed 7.83 ± 2.03	MedDiet adherence, weight, height, BMI, % body fat	5/8
Toti et al., 2022 [56] Italy	Wheelchair adult male basketballers, n = 15, age 28.5 ± 1.5 year; gym attendees, n = 15, age 31.5 ± 2.2 years; healthy non-active people, n = 15, age 30.1 ± 2.2 years		MDS 3-day diet record wheelchair basketballers 6.3 ± 0.6, gym attendees 7.5 ± 0.5, healthy non-active people 6.7 ± 0.5	Orthorexia nervosa, gastro-oesophageal reflux, neurogenic bowel dysfunction	8/8
**Experimental Studies**					
**Author** **Country of Study**	**Athlete Characteristics**	**Intervention and Study Design**	**Dietary Assessment and Monitoring of Adherence**	**Outcomes Assessed**	**JBI Score for Quality of RCT Study or Quasi-Experimental Study**
**Randomised Controlled Trials**					
Baker et al., 2019 [58] United States of America	Recreationally active athletes f—n = 7, m—n = 4 age 28 ± 3 year, BMI 24.6 ± 3.2 kg/m^2^	Randomised sequence cross-over trial. A total of 4 days of MedDiet and 4 days of Western diet. A 9–16-day washout period between 2 diets Not indicated if dietitians provided diets No specific sports nutrition recommendations provided	A 3 × 4-day weighed electronic dietary record during each diet period. Research dietitians monitored adherence and assessed food records Significant increase in intake of fruits, vegetables, fish, and olive oil	5 km treadmill time trial, Wingate cycle test, vertical jump test, handgrip strength, heart rate	10/13
Chilelli et al., 2016 [59] Italy	Male masters cyclists, n = 47	3-month RCT 2 groups: 1. MedDiet—age 46 ± 8 years, wt 71.8 ± 9.6 kg, BMI 23.0 ± 6.4 kg/m^2^ 2. MedDiet + curcumin (10 mg) + Boswellia (140 mg)—age 45 ± 9 years, wt 72.4 ± 8.0 kg, BMI 23.7 ± 2.1 kg/m^2^ No specific sports nutrition recommendations provided	FFQ—assessed by dietitians, adherence to MedDiet and intake not assessed	Oxidative stress, total advanced glycation endpoints (AGEs), inflammatory mediators	7/13
Ficarra et al., 2022 [62] Italy	Cross-fit athletes with 1 yr of regular training for a minimum 3 times/week n = 30, f—n = 15, m—n = 15, age 20–50 years	8-week RCT, MedDiet vs. Habitual Diet Diets planned by dietitian Protein intake based on training sessions and training load per week—range was 1.4–2.0 g/kg/d	Dietary intakes not assessed	Body composition, body circumferences, Wingate 30 s, quat jump, countermovement jump, 30 s jump, push-up test to exhaustion, chin-up test to exhaustion, Fran training	6/13
Malaguti et al., 2008 [60] Italy	Male, non-professional volleyball athletes who trained at least 3 times/week, and each training session lasted 2 h n = 11	2-month RCT MedDiet vs. High-protein low-caloric diet + fish oil diet No specific sports nutrition recommendations provided	Detailed record of weekly food intake (frequency not stated) FFQ—3 times during study Adherence to diet not reported Not stated if dietitians assessed food records	BMI Body composition, total antioxidant activity	6/13
Miralles-Amoros et al., 2023 [63] Spain	Elite female professional handball players n = 21, age 22 ± 4 years	12-week RCT with 3 diet arms: 1. Free diet. 2. MedDiet. 3. High-antioxidant diet.	7-day self-recorded dietary record at 3 timepoints Adherence not reported	Weight, height, BMI, Body composition, Eating behaviour (EAT-26), Body image BSQ, Mood (POMS)	8/13
Soldati et al., 2019 [61] Italy	Kickboxers Male n = 20 and Half-marathon runners Male n = 20 Athletes from all competition levels	3-month Nutritional counselling intervention on MedDiet vs. Control diet Sports-specific recommendations—considered primary energy source dependent on sports	3-day diary at beginning and end of study MedDiet adherence checked by food recall by nutritionists but not reported	Countermovement jump, squat jump, 15″ test, bench test, VO_2_ max, Body fat mass, RMR	7/13
**Quasi-experimental Studies**					
Caparello et al., 2023 [66] Italy	Male volleyballers from national league n = 11, age 19–37 years	All athletes underwent a nutrition education session and were provided with personalised plan before pre-season on MedDiet +/− ergogenic supplements by nutritionists Protein intake based on training sessions and training load per week—range was 1.0–1.5 g/kg/d	Weekly food diary MEDAS Not stated who checked food diaries Adherence at baseline 9.8 ± 1.1, pre-season 10 ± 0.2, season 10.3 ± 0.1, play-offs 10.2 ± 0.1 (all not significant)	MedDiet adherence, BMI, Body composition, Basal and Resting Metabolic rate	8/9
Philippou et al., 2017 [64] Cyprus	Adolescent competitive swimmers, f—n = 11, m—n = 23, and 22 parents) Mean age 15.2 ± 1.5 years	Nutrition education session on MedDiet (1/2 day)	KidMed Improvement in adherence with 47% having good adherence post-intervention vs. 21% at baseline (*p* < 0.01) and an increase in KIDMED Index score (median [interquartile range]: 5.0 [4.0–7.0] vs. 7.0 [7.0–9.0]; *p* < 0.01)	Nutrition knowledge, MedDiet adherence, changes in intakes of foods in MedDiet	8/9
Sahnoune et al., 2020 [65] Algeria	Adolescent athletes who train for 6–10 h/week in handball, athletics, basketball, swimming, judo	Nutrition education sessions on MedDiet (6 sessions). Changes assessed after 6 months	KidMed At baseline, 8% good, 31% moderate, 61% poor adherence After intervention, 60% good, 30% moderate, 10% poor adherence.	MedDiet adherence, weight, BMI, International physical activity questionnaire (IPAQ)	7/9

Abbreviations: BMI—body mass index; Wt—weight; MedDiet—Mediterranean Diet; m—male; f—female.

Two intervention studies assessed whether carbohydrate and protein intakes from the prescribed MedDiet met sports nutrition guidelines [4,71,73]. Adolescent athletes [65] consumed an average of 1.5 g/kg of body weight per day (BW/d) of protein with 50–60% derived from animal sources, thus meeting the minimum recommendation set for adult athletes [71]. The average carbohydrate intake was between 5 and 7 g/kg of BW/d across the six-month study, which also met sports nutrition guidelines for carbohydrates [73]. Wheelchair basketballers [56] achieved a mean intake of 3.6 g/kg of BW/d of carbohydrates and 1.5 g/kg of protein on the MedDiet.

The study outcome measures and results are mapped in Table 3 and Table 4. Anthropometric and body composition measures were the most frequently reported outcomes assessed in relation to the MedDiet in the included studies. BMI was the most commonly reported (n = 19 articles) [34,37,38,39,40,42,46,47,48,49,51,52,55,59,60,62,63,65,66], followed by body weight (n = 14) [34,38,39,42,46,47,48,49,51,55,59,62,63,65] and standing height (n = 8) [42,46,47,48,49,55,63,65]. Waist circumference was measured in three studies [46,49,62] as was the waist-to-hip ratio [34,35], and the leg and arm circumference was measured in one study [62]. The measures of body composition assessed by bio-electrical impedance analysis (BIA) were reported across 12 studies [36,38,42,47,48,53,54,59,60,61,62,66]. Only one study assessed body composition using DXA [35], and three studies used skinfold measures [49,55,63].

Overall, there was inconsistent evidence supporting a relationship between adherence to the MedDiet and anthropometric and body composition measures. The observational studies reported mixed results with no clear pattern related to the type of athlete, sport, gender, or age. Only one RCT, on cross-fit athletes [62], demonstrated significant increases in arm and leg circumference, and one quasi-experimental study showed increases in FFM in volleyball players after following a MedDiet intervention [66].

Numerous physical performance and fitness indicators were reported as outcomes in the observational studies. Strength and explosive power output was assessed by handgrip strength in six studies [36,37,39,40,48,50]; push strength and the range of shoulder motion [50] and the countermovement jump (CMJ) and Abalakov jump test were assessed in five studies [36,38,39,42,55], all of which presented conflicting findings. Romero-Garcia [55] also examined the relationship between MedDiet adherence and the T-half test, yo-yo test, squat jump, overhead medicine ball throw test, and the 30 m sprint; however, no associations were observed.

Only three observational studies [35,40,44] examined the relationship between MedDiet adherence and its direct relationship with sporting performance. No association was found between MedDiet adherence and 100 km running performance in Italian national ultramarathon runners [35]. Similarly, adherence to the MedDiet was not associated with the level of competition (as a proxy measure of performance) in adolescent Greek gymnasts aged 11–18 years [51] or in cyclists [44].

In the RCTs, the MedDiet led to some significant improvements in performance outcomes (Table 4). In kickboxers and runners, there were improvements in countermovement jumps, the 15-inch test, and squat jumps [61]. In cross-fit athletes, there were improvements in squat jumps, the Wingate cycle test, the push-up test, the chin-up test, and the Fran test [62]. Of the nine intervention studies, only one RCT [58] directly evaluated performance outcomes in athletes directly related to their sport (endurance running). They reported significant improvements in running velocity (5 km run time), with no difference in heart rate or perceived effort, after 4 days of the MedDiet intervention [58].

Few studies assessed health-related clinical outcomes. Physiological and biochemical parameters were assessed in five intervention studies; these parameters included heart rate [58], resting and/or basal metabolic rate [61,66], cellular fatty acids [60], inflammatory markers [59], and antioxidant activity [59,61]. In two experimental studies, differences were reported in the oxidative response in cyclists and volleyball players on the MedDiet. Chilelli et al. [59] reported significant reductions in some oxidative markers and a significant increase in the inflammatory marker TNFα, but not in IL-6, in masters cyclists. However, in volleyball athletes, no significant difference in total antioxidant activity between the MedDiet group and a high-protein, low-carbohydrate, fish oil-supplemented group was observed [61]. No change in heart rate [58] or resting metabolic rate following the MedDiet intervention was observed in kickboxing or half-marathon athletes [63]. One observational study analysed associations between MedDiet adherence and bone quality in female beach handball players, where no relationship observed [45]. One observational study [57] conducted on wheelchair basketballers examined the association between MedDiet adherence and neurogenic bowel dysfunction and found that increased adherence to the MedDiet was positively associated with the worsening of symptoms related to neurogenic bowel dysfunction; however, no relationship was observed with symptoms related to gastro-oesophageal reflux disease.

A range of mental health and wellbeing parameters were assessed in relation to performance and health such as anxiety [40], burnout syndrome [45], motivation to exercise [50], body image [49,63], and orthorexia nervosa [56,57]. Greater adherence to the MedDiet was inversely associated with burnout syndrome in younger tennis players [45]. In contrast, greater adherence to the MedDiet was positively associated with body dissatisfaction in female acrobatic gymnasts [49]. Adherence to the MedDiet was not related to anxiety in adult sailors or motivation to exercise in canoeists [40,50]. In professional athletes [57] and wheelchair basketball players [56], higher adherence to the MedDiet was significantly correlated with more pathological behaviours associated with orthorexia nervosa. Eating behaviours (assessed with the EAT-26, Body image (BSQ), and mood (POMS) scales) were examined in one RCT [63] in female professional handball players; however, no significant differences in any of these outcomes were observed.

### Assessment of Quality of Studies

Quality appraisal scores for all included studies are shown in Table 2. Most observational studies were assessed as medium-quality; four studies were identified as high-quality (8 out of 8) [40,53,56,57], and five studies [35,39,43,47,50] were identified as low-quality (≤5 out of 8). Low-quality studies did not account for confounding factors or define inclusion criteria. A detailed assessment of the quality of studies is in Supplementary Tables S2–S4.

The majority of the RCTs were assessed as low–medium-quality, with the most common concerns being the lack of participant and researcher blinding and an inadequate description of allocation concealment. Only one of six intervention studies was rated medium–high-quality and adequately described the randomisation and allocation concealment process [58]. The three quasi-experimental studies received high-quality appraisal scores. They did not use a control group; however, all three studies used a pre–post-test approach [64,65,66].

The quality of the design, management, composition, and prescription of the MedDiet interventions from the included RCTs and quasi-experimental studies is presented in Table 5. Overall, the quality of the prescription and reporting of all MedDiet interventions was poor. Only one RCT provided specified quantities of food groups consistent with the MedDiet pattern (Supplementary Table S5) and provided details on at least 8 of 19 pre-defined elements consistent with the MedDiet (including food, meal preparation, and socialisation with meals) [58]. Six of the intervention studies had the diets designed and administered by research dietitians/nutritionists [60,61,62,64,65,66]; however, it was unclear whether research personnel in the remaining studies were sports dietitians/nutritionists [58,59,63]. All of the six RCTs provided dietary information either through written or verbal education [58,59,60,61,62,63]; however, only two intervention studies reported providing individualised dietary counselling [61,63]. Two of the three quasi-experimental studies [59,66] provided individualised dietary counselling.

## 4. Discussion

To the best of our knowledge, this is the first scoping review to systematically map and synthesise the available literature on adherence to the MedDiet and outcomes related to exercise and sporting performance in elite or recreational athletes. Our mapping of the literature showed that anthropometric, performance, clinical, and psychological parameters were the major outcomes reported in the 23 observational and 9 intervention studies identified. Overall, our review highlights that there is limited evidence for the application of the MedDiet in an athletic population and, in particular, in any defined athlete group by sport, age, or gender. In particular, there are a limited number of robust RCTs evaluating a broad range of exercise and sporting performance outcomes, necessitating the need for more high-quality research to establish a higher level of causal evidence in order to guide prescriptive dietary advice and recommendations for the MedDiet in an athletic population. This will allow sports dietitians and nutritionists to make informed dietary recommendations based on evidence to support athletes in improving their performance. An important gap identified in the present scoping review is whether a prescriptive MedDiet would provide adequate carbohydrate and protein to meet current sports nutrition guideline requirements to optimise athletic performance and physique traits. Six of the nine intervention studies included in the present review did not provide individualised guidelines with respect to energy, carbohydrate, and protein requirements consistent with sports nutrition guidelines [58,59,60,61,62,65]. Of the three studies which included prescriptive recommendations, only two studies met the current recommendations with respect to carbohydrate and protein requirements. As such, there are currently limited data to support that the MedDiet would adequately fuel exercise and sporting performance. Furthermore, given that animal-based protein sources form a small component of the MedDiet, protein quality also needs to be considered, particularly in relation to the quantity of leucine and creatine. Leucine is an important branched-chain amino acid which is involved in stimulating muscle protein synthesis. However, the composition of leucine is limited in plant protein sources (beans, legumes, nuts) which are the key dietary components of the MedDiet [29,71]. Similarly, creatine, which plays a crucial role in the creatine phosphate energy system and is predominantly found in muscle meat, may be in limited supply in the MedDiet. Therefore, it is essential to evaluate creatine within the context of the MedDiet to inform recommendations regarding creatine supplementation [71].

In this review, we found no studies evaluating whether the MedDiet would suit the unique nutritional needs of masters athletes [74]. Given the significant body of evidence supporting the efficacy of the MedDiet in reducing the risks associated with chronic diseases [13] and sarcopenia [75], it would be beneficial to explore the implementation of the MedDiet in this set of athletes.

There are also limited studies that assessed the MedDiet in para athletes [76]. One study found more symptoms related to neurogenic bowel dysfunction in 15 wheelchair basketball athletes, perhaps related to the inherent fibre intake associated with the MedDiet [56]. Further studies are needed to understand whether the MedDiet is suitable to meet the unique needs of a variety of para athletes. Individual variations, particularly in relation to fibre content, may be needed to address the needs of para athletes [77].

Athletes may spend considerable time travelling, training, and competing in unfamiliar countries, necessitating planning to ensure the availability of the foods needed to maintain their training and performance diets [78]. This review did not find any studies which considered the feasibility and acceptability of adhering to the MedDiet in these situations.

Most of the observational studies in this review reported moderate adherence to the MedDiet, and of the intervention studies that measured adherence, all reported increases in adherence following the intervention. This is in agreement with a recent meta-analysis which reported small but significant increases in adherence in young people [79]. However, the long-term sustainability of adhering to the MedDiet has not been reviewed. 

The majority of the studies reported on in this review were conducted in Mediterranean countries, where the acceptability and adherence to the MedDiet are likely to be higher than those in non-Mediterranean countries. A recent narrative review [27] found that athletes have higher adherence to the MedDiet than the general population; however, all studies included in that review were also conducted in Mediterranean countries. Determining the acceptability of the MedDiet in athletes outside of Mediterranean countries is important; our previous work showed that Australian consumers perceive knowledge, motivation, access to food, affordability, time, and suitability as barriers to adopting the MedDiet [80]. Nevertheless, evidence from intervention studies included in this review suggests that MedDiet adherence can be increased in athletes. However, only two studies were conducted outside of the Mediterranean region [58,63]. As such, future studies should evaluate the feasibility, transferability, and acceptability of the MedDiet amongst athletes in non-Mediterranean countries and assess the benefits of adopting the MedDiet in contrast to local cultural diets to ensure that interventions are culturally appropriate and incorporate strategies aimed at improving culinary self-efficacy [81] and facilitating dietary compliance. Furthermore, this review highlights differences in the definition, composition, and interpretation of the MedDiet in research, with poor reporting making comparisons between studies difficult [16].

Athletes have identified health as an important determinant of food choice [82], so it is important to tailor dietary plans for athletes to address health and performance needs [4]. As such, investigating the potential health benefits of the MedDiet pattern for athletes is important; however, few studies in this review reported on health outcomes. One observational study reported no change in bone quality [39], and three intervention studies reported mixed outcomes with respect to changes in inflammatory markers, antioxidant capacity, and oxidative stress [59,60,61]. The MedDiet has several nutritive components (antioxidants, phenols, fibre, omega-3 fatty acids) which provide cardiometabolic and anti-inflammatory benefits. In addition to these health benefits, these nutrients may also prevent or reduce the risk of illness and injury while exercising [29]. Omega-3 fatty acids (found in fish and fish oil) have been shown to be provide potential therapeutic and prophylactic benefits in traumatic head injuries acquired in some sports [83,84]. Furthermore, a systematic review and meta-analysis of clinical trials involving omega-3 supplementation showed enhanced whole-body protein synthesis [85] in the general population. A recent systematic review showed that the MedDiet leads to higher levels of omega-3 fats in body tissues [86]. More research is needed to determine whether the MedDiet pattern will improve omega-3 fat levels in athletes and provide long-term prophylactic benefits, especially in masters athletes.

Strong, consistent relationships have been reported in the literature between MedDiet adherence and reductions in depressive symptoms [87,88] and improvements in cognitive health in older age [89,90], so there may be potential for the MedDiet to influence athlete mental health and performance-related cognition. There were few studies that examined athlete mental health in the present review, all with mixed outcomes. Further studies investigating the impact of the MedDiet on athlete cognitive performance and mental health are needed.

A strength of our review is that we used a comprehensive search strategy for the retrieval of articles, guided by a standardised framework. Furthermore, we also assessed the quality of each of the included studies against the JBI quality assessment tools and independently assessed the quality of MedDiet interventions. Nevertheless, a few limitations also need to be considered. Firstly, the classification of athletes into competitive, national, or amateur athletes may be inconsistent across the included studies as no reference was made to which classification system [91] was used by the individual studies [92]. Secondly, we limited studies to English only, which reduced the inclusion of studies from Mediterranean regions, particularly Spain. Additionally, the included studies in the present review had varying quality, especially RCTs. Concerns included pseudo-randomisation, the lack of allocation concealment, and the lack of the blinding of study participants and researchers to the dietary intervention. Quasi-experimental studies were of higher quality, but allocation concealment and randomisation were not assessed against the JBI appraisal checklist.

### Recommendations

This scoping review highlighted the challenges in determining the potential impact of the MedDiet on exercise performance outcomes due to variations in athletes (age, gender, level, and type of sport), outcome measures, and the designs of the included studies. Gaps in the evidence reflect the low number of studies, particularly intervention studies, thus providing very little high-quality evidence from which to draw conclusions about performance outcomes for specific sports or athlete groups. As such, several priorities for future research were identified:

1. Theoretical and intervention studies should consider the nutritional adequacy of the MedDiet for its potential to meet prescriptive sports nutrition guidelines (e.g., protein and CHO).
2. Theoretical and intervention studies need to consider the nutritional adequacy of the MedDiet for injury and rehabilitation—specifically looking at protein and other key micronutrients.
3. Future studies need to be conducted with athletes from non-Mediterranean countries to assess the adherence, feasibility, and acceptance of the MedDiet.
4. More high-quality intervention studies are needed that assess changes in anthropometric measures, sport-related performance outcomes, and wellbeing in a range of athletes (different ages and genders) to provide high-quality causal evidence.
5. A more rigorous reporting (both qualitative and quantitative) of the prescribed components of the MedDiet and adherence to the MedDiet is needed in future intervention studies.
6. Future studies should explore the feasibility and acceptability of the MedDiet for athletes while travelling for training and competition.
7. The nutritional adequacy, acceptability, and benefits of the MedDiet in masters athletes and para athletes need to be evaluated.
8. Future intervention and longitudinal observational studies should consider the impact of the MedDiet on short- and long-term health outcomes for athletes.

## 5. Conclusions

This scoping review identified several observational studies that reported sport and exercise performance outcomes related to MedDiet adherence amongst athletes; however, evidence from intervention studies is lacking. The poor reporting of MedDiet interventions limited the interpretation of the findings. The current evidence is inconclusive and does not support the widespread adoption of the MedDiet for athletes for exercise or sporting performance, though the MedDiet’s health benefits are well established. Priorities for future research include mapping the MedDiet against nutrient recommendations for athletes, assessing the acceptance of the MedDiet across varied athletic populations and settings, evaluating the benefit of the MedDiet for performance-related outcomes, and assessing the feasibility and benefits of the MedDiet for specific sports and athlete groups including para and masters athletes.

## Figures and Tables

**Table 1:** Summary of inclusion and exclusion criteria.

	Inclusion Criterion	Exclusion Criterion
Participants	Human studies with any competing, recreational, professional, elite, or occupational athlete (of any age) who plays a physical sport as part of a team or as an individual.	Studies which included participants involved in non-physical sports such as chess or video gaming
Intervention/exposure	Intervention studies which included a whole MedDiet intervention. Observational studies assessing adherence to the MedDiet using a Mediterranean dietary adherence tool. Studies assessing the delivery of single meals aligned with the MedDiet (acute feeding studies).	Studies administering only one or two components or constituents of the MedDiet (i.e., olive oil, nuts)
Comparisons	Intervention studies using any other type of dietary intervention or usual diet.	
Outcomes	Studies reporting on any outcome related to anthropometry, the assessment of body composition, or sporting or exercise performance or health outcome, e.g., cognitive performance, adherence to the MedDiet, sleep, burnout/fatigue.	Studies not reporting these outcomes
Study design	Any intervention study (RCT, pre–post-test series, open-label design RCT). Any observational study.	Systematic reviews and meta-analyses of intervention and observational studies. Conference abstracts, case studies

**Table 3:** Mapping of outcomes measured and results from included observational studies.

Author, Country, Athletes	Age	Sex	Weight	Height	Body Mass Index	Waist–Height Ratio	Waist Circumference	Arm/Leg Circumference	Body Composition	Performance/Success in Competition	Training Volume/MET Score	Range of Motion	20 m Shuttle Run Test	30 m Sprint	Countermovement Jump	Abalakov Jump	Squat Jump	Yo-yo Test	Medicine Ball test	T-half Test	Handgrip Strength	Push Strength	VO_2_ Max	Forced Vital Capacity	Forced Expiratory Flow	Peak Expiratory Flow	Body Dissatisfaction	Bone Quality	Motivation to Exercise	Burnout Syndrome	Anxiety	Obsession with Food	Risk Exercise Anxiety	Orthorexia Nervosa	Gastro-oesophageal Reflux	Neurogenic Bowel Dysfunction
Altavilla et al., 2021, Spain, Swimmers [34]	o	o	o	x	o	o	x	x	x	x	x	x	x	x	x	x	x	x	x	x	x	x	x	x	x	x	x	x	x	x	x	x	x	x	x	x
Citarella et al., 2021, Italy, Runners [35]	x	+ve ^f^	x	x	x	o	x	x	o	o	x	x	x	x	x	x	x	x	x	x	x	x	x	x	x	x	x	x	x	x	x	x	x	x	x	x
Kontele et al., 2021, Greece, Gymnasts [51]	x	x	−ve	x	−ve	x	x	x	x	x	x	x	x	x	x	x	x	x	x	x	x	x	x	x	x	x	x	x	x	x	x	x	x	x	x	x
Leao et al., 2023, Portugal, Soccer athletes [52]	x	x	x	x	o	x	x	x	x	x	x	x	x	x	x	x	x	x	x	x	x	x	x	x	x	x	x	x	x	x	x	x	x	x	x	x
Lopez-Jimenz et al., 2023, Spain, Rugby union athletes [53]	x	x	x	x	x	x	x	x	+ve ^p^	x	x	x	x	x	x	x	x	x	x	x	x	x	x	x	x	x	x	x	x	x	x	x	x	x	x	x
Manzanio-Carrasco et al., 2020, Spain, Athletes [36] all	x	x	x	x	x	x	x	x	x	x	x	x	+ve	x	o	x	x	x	x	x	o	x	x	o	x	x	x	x	x	x	x	x	x	x	x	x
boys	x	x	x	x	o	x	x	x	o	x	x	x	o	x	o	x	x	x	x	x	+ve	x	x	+ve	+ve	+ve	x	x	x	x	x	x	x	x	x	x
girls	x	x	x	x	o	x	x	x	o	x	x	x	o	x	o	x	x	x	x	x	o	x	x	o	o	o	x	x	x	x	x	x	x	x	x	x
Manzano-Carrasco et al., 2020, Spain Footballers [37]	x	x	x	x	x	x	x	x	x	x	x	x	o	x	x	x	x	x	x	x	o	x	x	x	x	x	x	x	x	x	x	x	x	x	x	x
Manzano-Carrasco et al., 2020, Spain, Athletes [38]																																				
boys	x	x	+ve ^g^	x	o	x	x	x	+ve ^g^	x	x	x	+ve	x	o	x	x	x	x	x	+ve	x	x	x	x	x	x	x	x	x	x	x	x	x	x	x
girls	x	x	o	x	o	x	x	x	o	x	x	x	o	x	+ve ^g^	x	x	x	x	x	o	x	x	x	x	x	x	x	x	x	x	x	x	x	x	x
Marques-Sule et al., 2022, Spain Canoe polo athletes [50]	x	x	x	x	o	x	x	x	o	x	o	o	x	x	o	x	x	x	x	x	o	o	x	x	x	x	x	x	o	x	x	x	x	x	x	x
Martinez-Rodriguez et al., 2018, Spain, Sailors [40,41]	x	o	x	x	+ve ^f^	x	x	x	x	o	x	x	x	x	x	x	xx	x	x	x	x	x	x	x	x	x	x	x	x	x	o	x	x	x	x	x
Martinez-Rodriguez et al., 2021, Spain, Beach handball athletes [39]	x	x	o	x	+ve	x	x	x	x	x	x	x	x	x	x	−ve	x	o	x	x	o	x	x	x	x	x	x	x	x	x	x	x	x	x	x	x
Martinez-Rodriguez et al., 2021, Spain, Beach handball athletes [42]																																				
Junior	x	x	−ve ^f^	o	o	x	x	x	o	x	x	x	x	x	o	x	x	x	x	x	−ve ^f^	x	x	x	x	x	x	x	x	x	x	x	x	x	x	x
Senior	x	x	o	o	o	x	x	x	o	x	x	x	x	x	o	x	x	x	x	x	o	x	x	x	x	x	x	x	x	x	x	x	x	x	x	x
Martinez-Rodriguez et al., 2022, Spain Beach handball athletes [48]	x	x	−ve	o	−ve	x	x	x	o	x	x	x	x	x	o	o	x	+ve	x	x	o	x	+ve	x	x	x	x	x	x	x	x	x	x	x	x	x
Martinovic et al., 2022, Croatia, Professional athletes [57]	x	x	x	x	x	x	x	+ve	x	x	x	x	x	x	x	x	x	x	x	x	x	x	x	x	x	x	x	x	x	x	x	+ve	x	x	x	x
Mayolas-Pi et al., 2017, Spain, Endurance cyclists [43]	x	x	x	x	x	x	x	x	x	x	x	x	x	x	x	x	x	x	x	x	x	x	x	x	x	x	x	x	x	x	x	x	o	x	x	x
Morales-Suarez-Varela et al., 2023 Spain, Basketball athletes [54]	x	x	x	x	x	x	x	x	x	x	x	x	x	x	x	x	x	x	x	x	x	x	x	x	x	x	x	x	x	−ve	x	x	x	x	x	x
Papdopolou et al., 2017, Greece, Cyclists [44]	x	x	x	x	x	x	x	x	x	o	x	x	x	x	x	x	x	x	x	x	x	x	x	x	x	x	x	x	x	x	x	x	x	x	x	x
Pelaez-Barrios et al., 2022, Spain, Gymnasts [49]	o	x	o	o	o	x	o	x	o	x	x	x	x	x	x	x	x	x	x	x	x	x	x	x	x	x	−ve	x	x	x	x	x	x	x	x	x
Peraita-Costa et al., 2020, Spain, Tennis athletes [45]	x	x	x	x	x	x	x	x	x	x	x	x	x	x	x	x	x	x	x	x	x	x	x	x	x	x	x	x	x	−ve	x	x	x	x	x	x
Romero-Garcia et al., 2022, Spain, Handball athletes [55]	x	x	−ve	−ve	−ve	x	x	x	−ve	x	x	x	o	o	o	o	o	o	o	o	x	x	o	x	x	x	x	x	x	x	x	x	x	x	x	x
Santana et al., 2019, Spain, Gymnasts [46]	x	x	+ve	+ve	+ve	x	+ve	x	x	x	x	x	x	x	x	x	x	x	x	x	x	x	x	x	x	x	x	x	x	x	x	x	x	x	x	x
Santos-Sanchez et al., 2021, Spain, Soccer athletes [47]	x	x	o	o	o	x	x	x	o	x	x	x	x	x	x	x	x	x	x	x	x	x	x	x	x	x	x	x	x	x	x	x	x	x	x	x
Toti et al., 2022 Italy, Wheelchair basketball athletes [56]	x	x	x	x	x	x	x	x	x	x	x	x	x	x	x	x	x	x	x	x	x	x	x	x	x	x	x	x	x	x	x	x	x	−ve	o	+ve

MET score—metabolic equivalents score; g—prepubertal only; f—females only; p—specific positions only. o—no relationship found; +ve—positive relationship; −ve—inverse relationship; x—not assessed.

**Table 4:** Mapping of outcomes measured and results from included experimental studies.

Author, Year, Country, Athletes	MedDiet Adherence	Nutrition Knowledge	Weight	Height	BMI	Waist Circumference	Body Composition	Leg/Arm Circumference	BMR/RMR	Success/Level	5 km Treadmill Time	Wingate Cycle	Countermovement Jump	Standing Jump	15″ Test	Bench Test	Squat Jump	Abalakov Jump	Handgrip Strength	Heart Rate	Vo2 Max	30 s Jump Test	Push-Up Test	Chin-Up Test	Fran Test	Cellular Fatty Acids	Antioxidant Activity	Immune Markers	Eating Behaviours	Body Image	Mood
Baker et al., 2019, US, Runners [58]	↑	x	x	x	x	x	x	x	x	↑	↑	o	o	x	x	x	x	x	o	o	x	x	x	x	x	x	x	x	x	x	x
Caparello et al., 2023, Italy, Volleyball athletes [66]	o	x	x	x	↑	x	↑	x	↑	x	x	x	x	x	x	x	x	x	x	x	x	x	x	x	x	x	x	x	x	x	x
Chilleli et al., 2016, Italy, Cyclists [59]	x	x	o	x	o	x	o	x	x	x	x	x	x	x	x	x	x	x	x	x	x	x	x	x	x	x	mo	mo	x	x	x
Ficarra et al., 2022, Italy, Cross-fit athletes [62]	x	x	o	x	o	o	o	↑	o	x	x	↑	o	↑	x	x	x	x	x	x	x	o	↑	↑	↑	x	x	x	x	x	x
Malaguti et al., 2008, Italy, Volleyball athletes [60]	x	x	x	x	o	x	o	x	x	x	x	x	x	x	x	x	x	x	x	x	x	x	x	x	x	o	o	x	x	x	x
Miralles-Amoros et al., 2023, Chile, Handball players [63]	o	x	o	o	o	x	o	x	x	x	x	x	x	x	x	x	x	x	x	x	x	x	x	x	x	x	x	x	o	o	o
Philippou et al., 2017, Greece, Swimmers [64]	↑	↑	x	x	x	x	x	x	x	x	x	x	x	x	x	x	x	x	x	x	x	x	x	x	x	x	x	x	x	x	x
Sahnoune et al., 2020, Algeria, Athletes [65] All	x	x	o	o	o	x	x	x	x	x	x	x	x	x	x	x	x	x	x	x	x	x	x	x	x	x	x	x	x	x	x
Male	↑	x	↑	↑	↑	x	x	x	x	x	x	x	x	x	x	x	x	x	x	x	x	x	x	x	x	x	x	x	x	x	x
Female	↑	x	↑	↑	o	x	x	x	x	x	x	x	x	x	x	x	x	x	x	x	x	x	x	x	x	x	x	x	x	x	x
Soldati et al., 2019, Italy [61] Kickboxers	x	x	x	x	x	x	↑	x	o	x	x	x	↑	o	o	o	↑	o	x	x	o	x	x	x	x	x	x	x	x	x	x
Runners	x	x	x	x	x	x	↑	x	o	x	x	x	↑	o	↑	o	o	o	x	x	↑	x	x	x	x	x	x	x	x	x	x

BMI = body mass index; g—prepubertal only; f—females only; o—no change; ↑—significant improvement, ↓—significant worsening; x—not assessed; mo—mixed significant outcomes.

**Table 5:** Quality rating of MedDiet intervention in RCTs and quasi-experimental studies from criteria listed in Supplementary Table S6.

Quality Indicator of MedDiet Intervention	Baker et al., 2019 [58]	Caparello et al., 2023 [66]	Chilelli et al., 2016 [59]	Ficarra et al., 2022 [62]	Malaguti et al., 2008 [60]	Miralles-Amoros et al., 2023 [63]	Philippou et al., 2017 [64]	Sahnoune et al., 2020 [65]	Soldati et al., 2019 [61]
Diet designed and administered by dietitian	N	Y	N	Y	Y	N	Y	Y	Y
Minimum amounts specified to participants for identified Mediterranean foods	Y	N	N	N	N	N	N	N	N
Prescribed diet meets at least 8 out of 19 defined minimum criteria (Supplementary Table S6)	Y	N	N	N	N	N	N	N	N
Diet tolerance reported	N	N	N	N	N	N	N	N	N
Frequency and setting for diet instruction reported	Y	N	N	N	N	N	Y	Y	N
Diet compliance assessed	Y	N	N	N	N	N	Y	Y	N
Perception of diet burden or benefit reported	N	N	N	N	N	N	N	N	N

N—no; Y—yes.

## Data Availability

No new data were created or analyzed in this study. Data sharing is not applicable to this article.

## Supplementary Materials

The following supporting information can be downloaded at https://www.mdpi.com/article/10.3390/nu16244259/s1, Table S1: Search strategies with different databases; Table S2: JBI critical appraisal checklist of Observational Studies; Table S3: JBI critical appraisal checklist of Randomized Controlled Trials; Table S4: JBI critical appraisal checklist for quasi-experimental studies; Table S5: Dietary Elements prescribed for the Mediterranean Diet; Table S6: Definitions for quality indicators for Table 5 in paper.
