# Supplementary material for: The Relationship Between the Mediterranean Dietary Pattern and Exercise and Sport Performance—A Scoping Review
Source: Nutrients. 2024 Dec 10;16(24):4259. doi: 10.3390/nu16244259 (PMC11678181; doi:10.3390/nu16244259)
Supplement: Supplementary file 1 [file nutrients-16-04259-s001.zip › nutrients-3316172-supplementary.pdf]

Supplementary Table S1. Search strategies with different databases.

Database	Search terms	Filters applied
**PubMed**	"Mediterranean diet" AND (sport* OR athlet*)	English, Humans
**CINAHL**	"Mediterranean diet" AND (sport* OR athlet*)	English, Human
**Scopus**	"Mediterranean diet" AND (sport* OR athlet*)	English, Human
**ProQuest**	"Mediterranean diet" AND (sport* OR athlet*)	English
**Web of Science**	"Mediterranean diet" AND (sport* OR athlet*)	English
**SPORTDiscus**	"Mediterranean diet" AND (sport* OR athlet*)	English

Supplementary Table S2. JBI critical appraisal checklist of Observational Studies

**Article**	**Q1**	**Q2**	**Q3**	**Q4**	**Q5**	**Q6**	**Q7**	**Q8**	**Total**
Altavilla et al., 2021[34]	Y	Y	Y	Y	N	NA	Y	Y	6
Citarella et al., 2021[35]	N	Y	Y	NA	UN	NA	Y	Y	4
Kontele et al., 2021[36]	Y	Y	Y	Y	N	NA	Y	Y	6
Leao et al., 2023[37]	Y	Y	Y	Y	Y	N	Y	Y	7
Lopez-Jimenez et al., 2023[38]	Y	Y	Y	Y	Y	Y	Y	Y	8
Manzano-Carrasco et al., 2020 [39]	Y	Y	Y	Y	N	NA	Y	Y	6
Manzano-Carrasco et al., 2020[40]	Y	Y	Y	Y	N	NA	Y	Y	6
Manzano-Carrasco et al., 2020[41]	Y	Y	Y	Y	N	NA	Y	Y	6
Marques-Sule et al., 2022[42]	Y	Y	Y	Y	UN	NA	Y	Y	6
Martínez-Rodríguez et al., 2018[43]	Y	Y	Y	Y	Y	Y	Y	Y	8
Martínez-Rodríguez et al., 2021[46]	Y	Y	Y	Y	N	NA	Y	Y	6
Martínez-Rodríguez et al 2021[45]	Y	Y	NA	Y	UN	NA	Y	Y	5
Martinez-Rodriguez et al., 2022[47]	Y	Y	Y	Y	N	N	Y	Y	6
Martinovic et al., 2022[48]	Y	Y	Y	Y	Y	Y	Y	Y	8
Mayolas-Pi et al., 2017[49]	N	Y	NA	N	Y	Y	Y	Y	5
Morales- Suarez-Varela et al., 2023[50]	Y	Y	Y	Y	N	NA	Y	Y	6
Papadopoulou et al., 2017[51]	Y	Y	Y	Y	Y	N	Y	Y	7
Pelaez-Barrios et al., 2022[52]	Y	Y	Y	Y	N	N	Y	Y	6
Peraita-Costa et al., 2020[53]	Y	Y	Y	Y	N	N	Y	Y	6
Romero-Garcia et al., 2022[54]	Y	Y	Y	Y	N	NA	Y	Y	6
Santana et al., 2019[55]	Y	Y	Y	Y	N	N	Y	Y	6
Santos-Sanchez et al., 2021[67]	Y	Y	Y	NA	N	NA	Y	Y	5
Toti et al., 2022[57]	Y	Y	Y	Y	Y	Y	Y	Y	8

Y- Yes, N- No, NA – Not applicable, UN - Unclear

Note: Q1 = Were inclusion criteria defined? Q2 = Were detailed descriptions of participants provided? Q3 = Was exposure (diet) measured in a valid and reliable way? Q4 = Were objective, standard criteria used for measurement of the condition? Q5 = Were confounding factors identified? Q6 = Were there strategies to deal with the confounding factors? Q7 = Were outcomes measured in a valid and reliable way? Q8 = Was appropriate statistical analysis used?

Supplementary Table S3. JBI critical appraisal checklist of Randomized Controlled Trials

Article	Q1	Q2	Q3	Q4	Q5	Q6	Q7	Q8	Q9	Q10	Q11	Q12	Q13	Total
Baker et al., 2019 [58]	Y	Y	Y	N	N	N	Y	Y	Y	Y	Y	Y	Y	10/13
Chilelli et al., 2016 [59]	N	UN	Y	N	N	N	Y	Y	Y	Y	N	Y	Y	7/13
Ficarra et al., 2022[60]	N	N	Y	N	N	N	N	N	Y	Y	Y	Y	Y	6/13
Malaguti et al., 2008 [61]	N	N	Y	N	N	N	UN	Y	Y	Y	N	Y	Y	6/13
Miralles-Amoros et al., 2023[62]	Y	Y	UN	N	N	UN	UN	Y	Y	Y	Y	Y	Y	8/13
Soldati et al., 2019[63]	N	N	N	N	N	N	Y	Y	Y	Y	Y	Y	Y	7/13
Miralles-Amoros et al., 2023[62]	Y	Y	UN	N	N	UN	UN	Y	Y	Y	Y	Y	Y	8/13

Note: Q1 = Was true randomization used for assignment of participants to treatment groups? Q2 = Was allocation to treatment groups concealed? Q3 = Were treatment groups similar at the baseline? Q4 = Were participants blind to treatment assignment? Q5 = Were those delivering treatment blind to treatment assignment? Q6 = Were outcomes assessors blind to treatment assignment? Q7 = Were treatment groups treated identically other than the intervention of interest, Q8 = Was follow-up complete and, if Nt, were differences between groups in terms of their follow-up adequately described and analyzed? Q9 = Were participants analyzed in the groups to which they were randomized? Q10 = Were outcomes measured in the same way for treatment groups? Q11 = Were outcomes measured in a reliable way? Q12 = Was appropriate statistical analysis used? Q13 = Was the trial design appropriate, and any deviations from the standard randomized controlled trial design (individual randomization, parallel groups) accounted for in the conduct and analysis of the trial?

Y- Yes, N- No, NA – Not applicable, UN - Unclear

Supplementary Table S4. JBI critical appraisal checklist for quasi-experimental studies

Article	Q1	Q2	Q3	Q4	Q5	Q6	Q7	Q8	Q9	Total
Caparello et al., 2023[64]	Y	Y	Y	N	Y	Y	Y	Y	Y	8/9
Philippou et al., 2017 [65]	Y	Y	Y	N	Y	Y	Y	Y	Y	8/9
Sahnoune et al., 2020[66]	Y	Y	Y	N	Y	N	Y	Y	Y	7/9

Note: Q1 = Is it clear in the study what is the 'cause' and what is the 'effect' (i.e. there is N confusion about which variable comes first)? Q2 = Were the participants included in any comparisons similar? Q3 = Were the participants included in any comparisons receiving similar treatment/care, other than the exposure or intervention of interest? Q4 = Was there a control group? Q5 = Were there multiple measurements of the outcome both pre and post the intervention/exposure? Q6 = Was follow up complete and if Nt, were differences between groups in terms of their follow up adequately described and analyzed? Q7 = Were the outcomes of participants included in any comparisons measured in the same way? Q8 = Were outcomes measured in a reliable way? Q9 = Was appropriate statistical analysis.

Y- Yes, N- No

Supplementary Table S5. Dietary Elements prescribed for the Mediterranean Diet[1]

		Baker et al., 2019[58]			Caparello et al., 2023[64]			Chilelli et al., 2016[59]			Ficarra et al., 2022 [60]			Malaguti et al., 2008[61]			Miralles-Amoros et al., 2023[62]			Philippou et al., 2017 [65]			Sahnoune et al., 2020[66]			Soldati et al., 2019[63]		
Dietary Element	Minimum criterion	Mentioned	Qty given	Met min	Mentioned	Qty given	Met min	Mentioned	Qty given	Met mini	Mentioned	Qty given	Met min	Mentioned	Qty given	Met min	Mentioned	Qty given	Met min	Mentioned	Qty given	Met min	Met min	Qty given	Met min	Mentioned	Qty Given	Met min
Olive oil	2tbsp (40mL)/d or as principal fat/oil in diet	✓	✓	✓	-	-	-	-	-	-	-	-	-	✓	-	-	✓	-	-	-	-	-	-	-	-	-	-	-
Other oil/fat	Max allowed: 10g/d (~2tsp)	✓	✓	✓	-	-	-	-	-	-	-	-	-	-	-	-	-	-	-	-	-	-	-	-	-	-	-	-
Nuts	3s/w (1s = 30 g)	✓	✓	✓	-	-	-	-	-	-	-	-	-	-	-	-	-	-	-	-	-	-	-	-	-	-	-	-
Legumes	2s/w (1s = 150g or 1c cooked or canned legumes)	✓	✓	✓	-	-	-	-	-	-	-	-	-	✓	-	-	✓	-	-	-	-	-	-	-	-	✓	✓	✓
Vegetables	2s at 2meals/d or 4s/d total 1s = ½c cooked (75g) or 1c raw	✓	✓	-	-	-	-	-	-	-	-	-	-	✓	-	-	✓	-	-	-	-	-	✓	-	-	✓	✓	✓
Sofrito	2 x w rich tomato sauce with onion/garlic simmered in EVOO	-	-	-	-	-	-	-	-	-	-	-	-	-	-	-	-	-	-	-	-	-	-	-	-	-	-	-
Fruit	2s/d (1s = 1 medium or 2 small pieces or 1c diced fruit); excludes FJ	✓	✓	✓	-	-	-	-	-	-	-	-	-	✓	-	-	✓	-	-	-	-	-	✓	-	-	✓	✓	✓
Grain foods	1–2s/meal (or 3–6s/d) of any, preferably wholegrain (1s = 1sl bread or ½c cooked pasta, rice, barley, bulgur, etc.)	-	-	-	-	-	-	-	-	-	-	-	-	-	-	-	✓	-	-	-	-	-	-	-	-	✓	-	-
Fish or shellfish	2 s/w unless vegetarian (1s = 1 small fillet or 1 small can or 200g shellfish)	✓	✓	✓	-	-	-	-	-	-	-	-	-	-	-	-	✓	-	-	-	-	-	-	-	-	✓	✓	✓
Poultry	Max allowed: 2s/w (1s = 80g cooked or 100g raw)	✓	-	-	-	-	-	-	-	-	-	-	-	-	-	-	✓	-	-	-	-	-	-	-	-	-	-	-

Red meat	<2s/w red meat	✓	✓	-	-	-	-	-	-	-	-	-	-	-	-	-	-	-	-	-	-	-	-	-	-	✓	✓	✓
Processed meat	<1s/w processed meat	✓	-	-	-	-	-	-	-	-	-	-	-	-	-	-	-	-	-	-	-	-	-	-	-	✓	✓	✓
Eggs	Max allowed: 4/w	-	-	-	-	-	-	-	-	-	-	-	-	-	-	-	-	-	-	-	-	-	-	-	-	-	-	-
Dairy products	Max allowed: 2s/d 1 = ½c (120g) ricotta/cottage, 50g feta, ¾c (200g) yogurt, 2 slices (40g) cheese, 1c (250mL) milk	-	-	-	-	-	-	-	-	-	-	-	-	-	-	-	-	-	-	-	-	-	-	-	-	-	-	-
Sweets	Max allowed: 2s/w	✓	-	-	-	-	-	-	-	-	-	-	-	-	-	-	-	-	-	-	-	-	-	-	-	-	-	-
Sweetened drinks	Max allowed: <1s/d [1s = 1c (250 mL)	✓	✓	✓	-	-	-	-	-	-	-	-	-	-	-	-	-	-	-	-	-	-	-	-	-	-	-	-
Other discretionary	Max allowed: <2 s/w	-	-	-	-	-	-	-	-	-	-	-	-	-	-	-	-	-	-	-	-	-	-	-	-	-	-	-
Wine	Max allowed: 14 std drinks/w (1 std drink = 100 mL)	✓	✓	✓	-	-	-	-	-	-	-	-	-	-	-	-	-	-	-	-	-	-	-	-	-	-	-	-
Water	5c/d	-	-	-	-	-	-	-	-	-	-	-	-	-	-	-	-	-	-	-	-	-	-	-	-	-	-	-
Wet cooking methods	5c/d	-	-	-	-	-	-	-	-	-	-	-	-	-	-	-	-	-	-	-	-	-	-	-	-	-	-	-
Company when eating	≥4 main meals/w	-	-	-	-	-		-	-	-	-	-	-	-	-	-	-	-	-	-	-	-	-	-	-	-	-	-

1 – EVOO, extra virgin olive oil; d, day; w, week; s, serves; FJ, fruit juice; Max, maximum; Mentioned, element mentioned in study; Met min., minimum criterion for traditional diet was met; Qty given, quantity was specified by authors; std, standard; tbsp, tablespoon; tsp, teaspoon.

Supplementary Table S6. Definitions for quality indicators for Table 5 in paper.

Criterion 1	This criterion is satisfied if the report specifically mentions that the Mediterranean diet was both designed by a dietitian and participants were instructed by a dietitian on how to follow this diet.
Criterion 2	Specific daily/weekly quantities are stated for most of the Mediterranean foods recommended. This criterion is Nt met if only general guidance is provided to include more of certain Mediterranean foods or less of Nn-Mediterranean foods without instruction on minimum amounts required for consumption of the Mediterranean foods.
Criterion 3	When at least 40% of the minimum criterion* for 19 Mediterranean foods/cuisine aspects are met by the experimental diet.
Criterion 4	The study reports any intolerances to specific Mediterranean diet foods or the prescribed Mediterranean diet as a whole.
Criterion 5	The frequency or number of dietary instruction sessions provided during the study and the setting for these sessions (e.g. individual or group) is reported.
Criterion 6	Dietary compliance to the Mediterranean diet has been assessed in some way, at any time point, during the trial. This may include food records, FFQ and indices.
Criterion 7	The study collected data from participants on their perception of burden or benefit for the Mediterranean diet.
